# The Equine Gingiva: A Gross Anatomical Evaluation

**DOI:** 10.3389/fvets.2019.00322

**Published:** 2019-10-04

**Authors:** Saskia Steinfort, Carmen Obach-Schröck, Michael Röcken, Felix Theiss, Klaus Failing, Jörg Vogelsberg, Carsten Staszyk

**Affiliations:** ^1^Faculty of Veterinary Medicine, Institute of Veterinary Anatomy, Histology and Embryology, Justus-Liebig-University Giessen, Giessen, Germany; ^2^Clinic of Equine Surgery, Faculty of Veterinary Medicine, Justus-Liebig-University Giessen, Giessen, Germany; ^3^Vetsuisse Faculty, Equine Hospital, University of Zurich, Zurich, Switzerland; ^4^Unit for Biomathematics and Data Processing, Faculty of Veterinary Medicine, Justus-Liebig-University Giessen, Giessen, Germany

**Keywords:** gingival margin, gingival sulcus, horse, interdental papilla, periodontium

## Abstract

Equine periodontal disease (ePD) usually starts with food impaction, formation of diastemata, gingival inflammation and formation of periodontal pockets. This process proceeds toward the dentoalveolar space, causing detachment of tooth supporting periodontal fibers. Although several therapeutical procedures have been proposed, ePD is often only diagnosed in advanced stages, requiring dental extraction. A similar dilemma has been observed in small animal medicine, but has been overcome by the introduction of reliable examination protocols for the early diagnosis of periodontal diseases (PD). These protocols are based on detailed anatomical descriptions of healthy gingiva, allowing for the determination of the pathognomonic signs of the onset of PD and providing a basis for grading systems and treatment plans. Consequently, proposals have also been made for periodontal examination protocols in horses. However, these protocols were widely adopted from small animal medicine assuming a similar anatomy of the equine and canine gingiva. To provide a solid anatomical basis for equine specific periodontal examinations, 20 equine heads were examined macroscopically, with special attention to the gingival sulcus, the gingival margin and the interdental papillae. Constant morphological patterns of the gingival margin and the interdental papillae were found for the vestibular and lingual/palatal aspects of the upper and lower cheek teeth arcades, as well as for the incisor arcades. A gingival sulcus measuring greater than 1 mm was present in only 6% of the investigated specimens. The inspection of the gingival margin and the interdental papillae, as well as the recognition of a gingival sulcus, may serve as criteria to establish equine specific periodontal investigation protocols.

## Introduction

Multiple and detailed descriptions of the gingival anatomy of brachydont species exist (1–5). Erupted teeth are surrounded by the gingiva in a collar-like fashion (6–8). The gingiva can be divided into two parts according to its position in relation to the teeth, i.e., the interdental gingiva and the non-interdental gingiva. The non-interdental gingiva is located buccally/labially and palatally/lingually to the teeth. At the mucogingival junction (MGJ), the non-interdental gingiva merges with the oral mucosa (9). The non-interdental gingiva is further divided into the attached gingiva, which is tightly adhered to the periosteum of the alveolar margin, and the unattached gingiva, which rises above the alveolar margin and surrounds the tooth. At the gingival groove, the attached gingiva merges into the unattached gingiva. The gap between the unattached gingiva and the neighboring tooth is called the gingival sulcus (2, 3, 10). Although the equine gingival sulcus has been assumed to have a depth of up to 5 mm under healthy conditions (11), no morphometric analysis or descriptive studies have been conducted to date.

The interdental gingiva is located between adjacent teeth. It is bordered buccally/labially and lingually/palatally by the interdental papilla (IP) (1). Healthy human interdental gingiva have been described as two different shapes, depending on its localization: a pyramidal shape, when the occlusal tip of the gingiva lies directly under the contact point of two adjacent teeth; and a ‘col' shape, when the occlusal border of the interdental gingiva has a concave shape, with its tips at the buccal/labial and palatal/lingual end. The shape type depends on the manner in which the adjoining teeth are in contact with each other (2).

To the authors' knowledge, to date, there is no detailed description of the gingival anatomy in horses, including the interdental gingiva, the IP, the gingival sulcus, the MGJ, etc. As the hypsodont equine dentition features very tight contact between neighboring teeth, an equine-specific gingival anatomy is assumed, showing marked differences, compared to the well-described gingival anatomy of brachydont species. Thorough knowledge of the anatomy of the healthy equine gingiva is a prerequisite to diagnose the onset of gingival pathology.

Therefore, the aim of this study was to describe the gross anatomy and define specific landmarks of the equine gingiva, as a basis for the future development of reliable equine-specific periodontal grading systems.

## Materials and Methods

Twenty horses of different breeds, aged from <9 months to 26 years, were euthanized for reasons not related to this study (Table 1). Their heads were separated from their bodies and the jaws were dissected with a band saw (K440H, Kolbe Foodtec, Elchingen) in the location of the temporomandibular joint. Subsequently, the tongue was removed and all teeth were cleaned of loose food and debris.

**Table 1:** Data of horses examined.

**No**.	**Sex**	**Breed**	**Age**	**Cause of euthanasia**
1	Mare	Warmblood	9 months	Colic
2	Mare	Warmblood	6 years	Neoplasia
3	Mare	Warmblood	7 years	Colic
4	Mare	Warmblood	8 years	Colic
5	Mare	Warmblood	8 years	Colic
6	Gelding	Warmblood	10 years	Colic
7	Mare	Paint horse	11 years	Gastric ulcer
8	Mare	Warmblood	11 years	Colic
9	Gelding	Warmblood	12 years	Colic
10	Gelding	Pony	15 years	Laminitis
11	Mare	Appaloosa	16 years	Colic
12	Mare	Warmblood	17 years	Colic
13	Mare	Warmblood	18 years	Colic
14	Mare	Warmblood	19 years	Colic
15	Mare	Warmblood	20 years	Neoplasia
16	Gelding	Warmblood	20 years	Laminitis
17	Gelding	Paso peruano	21 years	Colic
18	Gelding	Arabian thoroughbred	23 years	Colic
19	Gelding	Warmblood	26 years	Urolithiasis
20	Mare	Warmblood	26 years	Colic

In each tooth position (P) of the dental arcade, the adjacent gingiva was inspected on the vestibular and palatal/lingual side. In total, there were 2,849 observed positions (OP). In preliminary investigations the intended measurements were tested and established in fresh cadaveric heads (<1 h after euthanasia), in frozen and thawed heads as well as in formalin-fixed specimens. Within the graduation we used (1 mm) no significant differences between the specimen were obtained. Therefore, the present study was performed using fresh, frozen and thawed as well as formalin-fixed heads. The following parameters were inspected.

### Gingival Sulcus

The presence of a gingival sulcus was assessed with the use of a blunt measuring probe (5 × 0.3 × 120 mm) with a 1-mm scale. The depth was assessed at the deepest point. The following categories were defined:

0: sulcus depth 0 mm to < 1 mm;
1: sulcus depth 1 mm to 2 mm;
2: sulcus depth > 2 mm to < 3 mm;
3: sulcus depth ≥ 3 mm.

### Periodontal Pocket

Periodontal pockets (PPs) were defined in cases in which the gingiva was not firmly attached to the tooth, but when an obvious gap between the tooth and the gingival margin was macroscopically visible and/or food particles were present between the gingiva and tooth. The border between the physiological gingival sulcus and the pathologic PP was marked by an abrupt sulcular deepening. The PPs were further evaluated with the use of a blunt measuring probe (Figure 2). Location, depth and length of the PPs were recorded according to Cox et al. (12).

**Figure 1:**
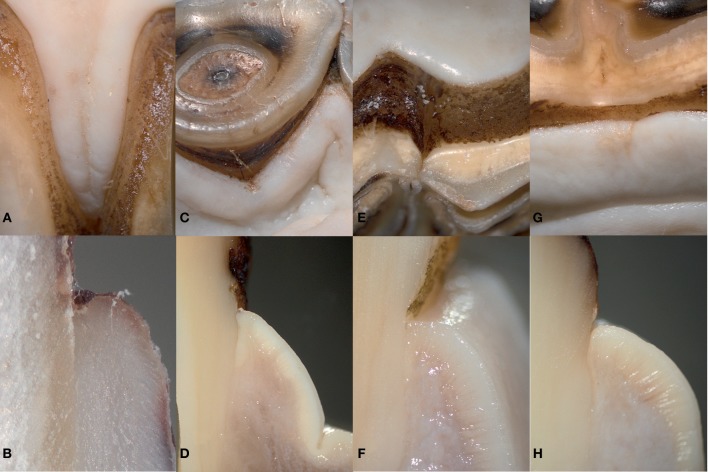
The equine gingiva is tightly attached to the tooth. **(A,C,E,G)**: macroscopic appearance of the equine gingiva in the incisor **(A,B)** and cheek teeth region **(E,G)**. **(B,D,F,H)**: corresponding transverse section. The gingiva is directly attached to the dental cementum featuring a gingival sulcus of less than 1 mm.

**Figure 2:**
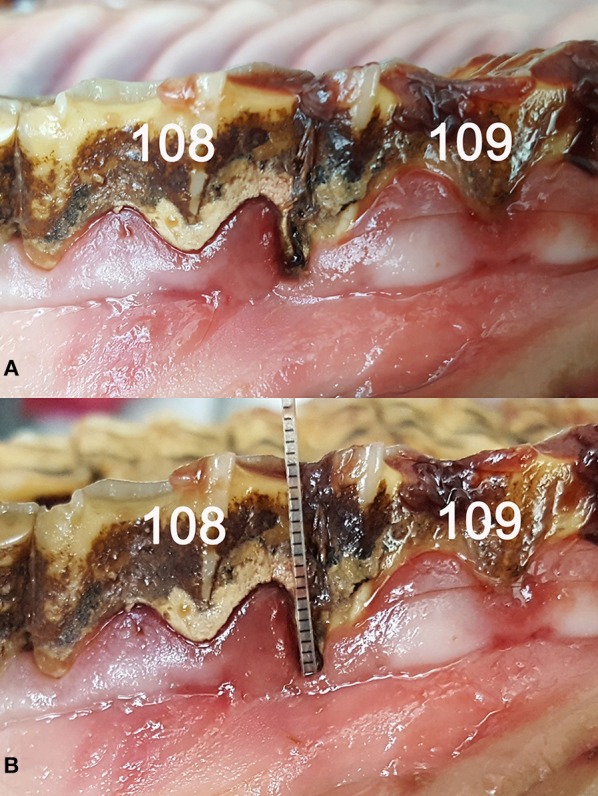
**(A)** Periodontal pocket between cheek teeth 108 and 109. **(B)** The inserted probe demonstrates the depth (5 mm) and position of the periodontal pocket.

### Diastema

Any interproximal gap between two neighboring teeth, containing or not containing food materials, was defined as a diastema.

### Mucogingival Junction

**Figure 3:**
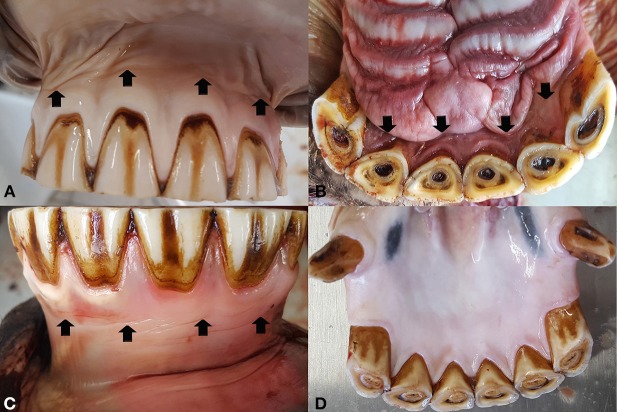
The mucogingival junction (arrows) of the incisors is clearly visible in the labial **(A)** and palatal **(B)** aspect of the upper incisors as well as in the labial aspect of the lower incisors **(C)**. In the lingual aspect of the lower incisors **(D)** the gingival fuses with the oral mucosa without a pronounced mucogingival junction.

**Figure 4:**
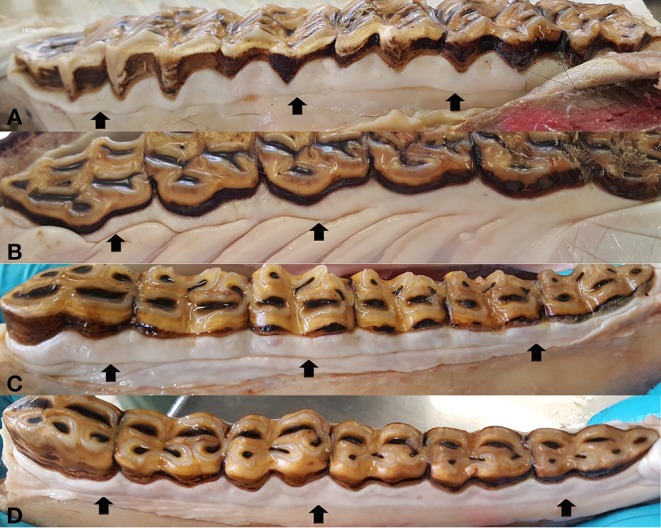
Mucogingival junction (arrows) in the cheek teeth, represented by an almost straight line in the buccal aspect of the upper cheek teeth **(A)**, buccal **(C)**, and lingual **(D)** aspect of the lower cheek teeth. In the palatal aspect of the upper cheek teeth **(B)**, the mucogingival junction fuses with the rugae palatinae.

The mucogingival junction (MGJ) was identified, according to the definition of (1), as the border between the firmly bound gingiva and the movable alveolar mucosa or the floor of the mouth mucosa (Figures 3, 4). The MGJ was categorized as present, less pronounced or absent.

### Shape and Contour of the Gingival Margin/Presence of a Papilla

**Figure 5:**
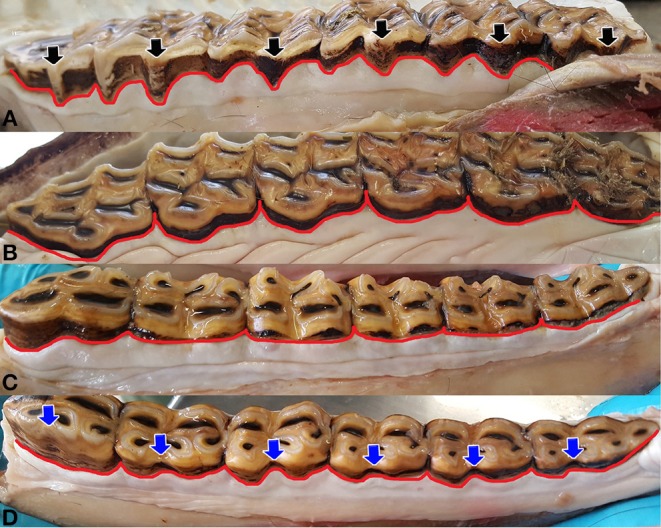
Shape and contour of the gingival margin (red line). The regularly double-waved contour of the buccal aspect of the upper jaw **(A)**, is composed of the pre- and post-mesostyle papilla, enclosing the mesostyle (black arrow). Interdental papilla can be seen irregularly. The palatal aspect of the upper jaw **(B)** shows a slightly undulatory single-waved contour, which can feature a marked interdental papilla. Shape and contour of the gingival margin, buccally **(C)** and lingually **(D)**, in the lower jaw, is almost straight. In teeth, with a pronounced linguaflexid (blue arrow), a dental papilla was additionally visible.

The shape and contour of the gingival margin (SCGM) adjacent to a tooth was analyzed and assigned to one of the following categories (Figure 5):

Almost straight;
Slightly undulatory;
Regular undulatory;
Irregular.

The SCGM is also formed by the presence or absence of the gingival papillae. Gingival papillae were defined as a protrusion of the gingival margin in the occlusal direction. Different positions of the gingival papillae were defined, using characteristic morphological structures of the equine tooth, i.e., the parastyle, which is a prominent enamel folding at the buccomesial aspect of the equine upper cheek teeth and the mesostyle, which is an enamel folding distal to the parastyle (Figure 5). The linguaflexid is an enamel folding at the lingual aspect of the equine lower cheek teeth:

*Interdental papilla:* portion of the gingiva that obviously entered the interdental space;
*Pre-mesostyle papilla:* gingival protrusion at the non-interdental aspects of the teeth in a pre-mesostyle position;
*Post-mesostyle papilla:* gingival protrusion at the non-interdental aspects of the teeth in a post-mesostyle position.

The presence or absence of the different papillae were recorded.

The statistical analyses were performed by means of the statistical program package R (Free Software Foundation's GNU project, official homepage: http://www.r-project.org) using the function glmmPQL within the package MASS and calling the package nlme. To analyse the frequency and/or strength of the different criteria, in dependency of the statistical scale of the target variable and the design of the experiment, a generalized linear mixed model analysis with a partial hierarchical design was performed, including the fixed effects of triadan quadrant, type of tooth (incisors or cheek teeth) and localization at the tooth (labial/buccal, lingual/palatal), as well as the random effects of the horse and the tooth within the horse. In addition, the age of the horse was used as a covariate in the model. For qualitative effects with more than two levels, a Wald test was carried out subsequently. For the description of the data, absolute and relative frequencies were computed. In all cases, a significance level of α = 0.05 was used.

## Results

### Gingival Sulcus

All Ops showed a gingival sulcus depth of less than 3 mm. In more than 90% (1,204/1,286), the depth was less than 1 mm (incisors: 99.7% [369/370]; cheek teeth: 91.2% [835/916]; *p* < 0.0001) (Figure 1). In the incisors, there was no difference in the presence of a gingival sulcus <1 mm between the palatal/lingual and buccal side of the teeth. In the upper cheek teeth, gingival sulci ≥1 mm were more frequently found on the palatal (4.3% [39/916]) than the buccal (1.6% [15/916]) side. In the lower cheek teeth, a greater number of gingival sulci ≥1 mm were found buccally (2.6% [24/916]) than lingually (0.3% [3/916]) (Table 2).

**Table 2:** Results of the glmm-analysis.

**Criterion**	**Type of tooth (incisors vs. cheek teeth)**		**Age**		**localization at the tooth (buccal vs. lingual/palatal)**		**Triadan quadrant**	**Triadan tooth number**
	***p*-value**	**OR**	***p*-value**	**OR**	***p*-value**	**OR**	***p*-value**	***p*-value**
Sulcus	<0.0001	0.027	0.48	0.95	0.75	1.1	<0.0001	
	Incisors		–	–	–	–	–	–
	Cheek teeth		0.47	0.95	0.90	1.025	<0.0001	0.022
Periodontal pocket	<0.0001	0.063	0.025	1.062	0.99	–	0.26	
	Incisors		–	–	–	–	–	
	Cheek teeth		0.037	1.065	0.92	–	0.32	0.0014
Muco gingival junction	<0.0001	0.004	0.0025	1.12	<0.0001	–	<0.0001	
	Incisors		0.014	1.12	<0.0001	–	<0.0001	<0.0001
	Cheek teeth		–	–	–	–	–	–
Papilla	<0.0001	84.2	0.018	0.94	0.0031	1.61	0.18	
	Incisors		–	–	–	–	–	–
	Cheek teeth		0.017	0.94	0.0005	1.71	0.21	0.13

*Incisors: sulcus, periodontal pocket, papilla: number too small; Mucogingival junction: number too small; Localization at the tooth, Triadan quadrant and Triadan tooth number: no single OR determined*.

### Periodontal Pocket

All horses had at least one PP. Altogether, PPs were found in 9.3% (145/1563) of all OPs. All PPs were visible without manipulation with the probe, and 75.9% (110/145) were correlated with food entrapment and/or diastemata. Of all the PPs, 85% (123/145) had a depth between 1 and 5 mm; the remaining 15% (22/145) showed a depth of more than 5 mm. Every assessed PP was found in an interproximal location. There were significantly (*p* < 0.0001) more PPs in cheek teeth (13.1% [141/145]) than in the incisors (0.8% [4/145]). The prevalence of PPs was similar in all interproximal spaces (*p* > 0.05). The occurrence of PPs in the cheek teeth significantly increased with age (*p* = 0.025).

### Diastema

In 11.4% (74/649) of the locations, a diastema was found. Diastemata were most frequently found in the position between Triadan 107/108, 306/307, and 308/309. In 41.9% (31/74), there was a correlation between diastemata, food entrapment and PPs. The premolar and molar regions showed more diastemata (14.3% [66/461]) than the incisor regions (4.3% [8/188]). Fewer diastemata were found in the lower jaw (incisors: 0% [0/98]; cheek teeth: 13.1% [30/229]) than in the upper jaw (incisors: 8.9% [8/90]; cheek teeth: 15.9% [37/232]). The presence of diastemata significantly increased with age (*p* = 0.03).

### Mucogingival Junction

The MGJ was grossly defined in the majority of cheek teeth (88.1% [2,536/2,878]), with no side- or jaw-related differences. Palatally, the MGJ merged into the rugae palatinae. The upper incisors also showed a constant presence of a MGJ on both sides (labial/palatal) in 90.9% (411/452) of the cases. In the lower incisors, the MGJ was present in 71.1% (150/211) of cases at the labial aspect, but was not evident (56.9% [120/211]) or less pronounced (43.1% [91/211]) at the lingual aspect (Figures 3, 4).

### Shape and Contour of the Gingival Margin/Presence of a Papilla

The gingiva of the incisors undulated in apical and occlusal direction in regular intervals (garland-shaped) at all localizations (palatal/lingual or buccal/labial) in both jaws, frequently showing an IP (98.6% [165/370]). The contour of the gingival margin in the cheek teeth varied between the localizations (upper vs. lower jaw, palatal/lingual side vs. buccal side).

The buccal aspect of the upper cheek teeth showed a regularly undulatory contour (Figure 5A), featuring a “double-waved” contour composed of a pre- and post-mesostyle dental gingival papilla. The post-mesostyle papilla was in continuation with an IP in 37.2% of the cases (71/191). The gingival margin of the upper cheek teeth on the palatal side showed a slightly undulatory, single-waved contour, featuring a marked IP, which was noted in 59.2% (113/191) of these OPs (Figure 5B).

In the lower jaw, the contour on both sides was almost straight. IPs were present in 55.3% (209/378; Figures 5C,D). In teeth showing a pronounced linguaflexid [see (13)], a papilla was additionally visible.

## Discussion

To the authors' knowledge, to date, no publications have focused on equine gingival anatomy. However, a few authors have investigated some aspects of the gingival anatomy while discussing the equine periodontium (9–11, 14).

In this study, in more than 90% of the OPs, no macroscopically visual sulcus was observed. Accordingly, most of the sulci had a measured depth of less than 1 mm. No sulcus was deeper than 3 mm. In the literature, sulcus depths under healthy conditions have been reported for several species. In human medicine, the gingival sulcus is described as a depth of 0.0–0.7 mm (15, 16). The canine sulcus depth is described to be <3 mm and the feline depth is <0.5 mm (17). Some authors have described a physiological equine sulcus depth of <5 mm (11, 12, 18), but do not provide any explanation as to how this value was obtained. However, for suggested equine gingival index systems (11, 12), a physiological gingival sulcus depth of <5 mm has been assumed. Based on the results of our study, we suggest a correction to <1 mm.

The specimens used in our study could not be examined for signs of inflammation *in vivo* (e.g., heat, redness, swelling). In future research, we strongly suggest *in vivo* measurements to verify the results of this study, as the vital unattached gingiva might show an increased height due to the physiological tissue tonus, and therefore a deeper gingival sulcus. However, marked differences between our cadaver study and *in vivo* measurements are not expected. All measurements were performed on non-fixed specimens and therefore only minor amounts of tissue alterations can be assumed.

The clear distinction between a PP and the physiological gingival sulcus is mandatory to distinguish between normal anatomical variation and a pathological condition. In the literature, the PP is defined as a pathologically-deepened sulcus (19, 20) and thus, the mere determination of its depth is an inappropriate criterion to identify a PP. We defined a PP by a macroscopically visible gap between the tooth and the gingival margin and by an abruptly increasing sulcular depth compared to adjacent parts of the gingiva.

The results of this study confirm the investigations of Cox et al. (12), who also found PPs located exclusively interproximally. Controversially, in human dentistry, PPs are described to possibly emerge everywhere throughout the gingival margin (19). Similarly, in canine medicine, there is no description of a particular localization of PPs. Thus, PPs in brachydont species are found in different localizations and are not exclusively linked to the interproximal regions, as in hypsodont equine dentition.

Accordingly, the etiopathogenesis of PP formation differs between horses and other species. In horses, PP formation has been correlated with malocclusion and the formation of gaps in the interproximal position (21). Food impaction, with the underlying formation of diastemata, results in a change of the bacterial flora. The progression of PD is instigated and the subsequent development of PP likely develops (22–24). Remarkably, horses are usually not affected by periodontal pocketing and PD at the sides of the teeth, but almost exclusively within the interproximal spaces. A possible explanation might be the very dynamic and regenerative features of the equine gingiva, which are forced to remodel continuously due to livelong tooth eruption.

In human dentition, a diastema is defined as a gap between two adjacent teeth (25). However, ungulates feature a large physiological interdental space between the incisors and premolars, which has also been referred to as a diastema in the veterinary anatomical nomenclature (26). To avoid misunderstandings, we suggest using the term diastema (pl. diastemata) exclusively for pathological conditions and using the alternative term margo interalveolaris for the physiological gap between incisors and premolars in ungulates.

In this study, 89% of the existing diastemata were found between cheek teeth. Only 11% were localized in the incisors. Results coincide with current literature, in which diastemata are mainly described in the cheek teeth region (27–29). There were inconsistencies in the main localization of diastemata. Although in this study more cheek teeth diastemata were located in the upper jaw, in the literature, most of the diastemata were found in the lower jaw (27, 30, 31). This is justified by the fact that the greatest masticatory forces, which contribute to diastemata, are generated in the caudal mandibular location (32). With an increased number of cases, this tendency might have also been seen in the present study. Due to the fact that the presence of diastemata is closely linked to PD (33, 34), practitioners should pay attention to this pathological change, with examination of the gingiva, too.

‘Diastema-linked PD' should be introduced in equine dentistry as a separate pathogenesis of PD in hypsodont species. In contrast to brachydont species, where PD is usually initiated by the formation of bacteria and plaque (35, 36), in the horse, PD commonly starts with diastemata formation. Afterwards, food gets impacted in the interproximal space. The destruction of adjacent gingiva, which functions as a barrier, and bacterial growth is the result. The emerging gingivitis can lead to pocket formation, gingival ulceration and cemental destruction, so that food impactions can get deeper into the interproximal space, promoting progressive inflammation and tissue destruction as a PD (30, 31, 37).

The MGJ is an important landmark in canine and human dentistry. It is not only used as a landmark in surgical procedures, but also important for measuring the gingival width and/or gingival recession, which may allow for an assessment of periodontal health status (1, 3, 38–41). In human and brachydont dentition, the MGJ is present, regardless of the region (cheek teeth or incisors/vestibular or lingual/palatal) (1, 3). In horses, the MGJ has also been described (9, 11) and recommended as a landmark for surgical procedures (42). However, so far, the MGJ has not been considered an indicator for gingival health in the horse. Several equine specific features might complicate a potential use of the MGJ in the horse and the assessment of gingival recession. First, the MGJ is not visible in all locations of the equine dentition, as documented in our data. Second, as the hypsodont equine tooth is a very dynamic structure, showing continuous tooth eruption. Therefore, the length of the clinical crown cannot be used as an landmark to monitor gingival recession. Third, in brachydont species, exposure of the cementoenamel junction is used as a criterion to classify gingival recession. As the entire equine tooth is covered by cementum a cementoenamel junction is absent and thus not applicable to determine gingival recession. However, future clinical studies are required to evaluate the possible use of the MGJ as a criterion to determine equine periodontal status.

SCGM plays an important role in the assessment of gingival health. Without the knowledge of the physiological SCGM, alterations (e.g., gingival recession or PPs) are rarely perceived. In human dentistry, the SCGM is described as parallel, following the undulating shape of the cementoenamel junction (1, 2). The embrasures of the tooth surface, the shape of the teeth, the subsequent shape of the gingival papilla and their alignment in the arch are substantially involved in the SCGM (2). Currently, there are no published descriptions of the SCGM in the dog and the horse. In this study, the SCGM varied within the locations, but, as described in human dentistry by Fiorellini (2), it nevertheless followed the embrasures of the tooth surface, the shape of the teeth, the subsequent shape of the gingival papilla (if present) and their alignment in the arch. In contrast to the partial absence of the IP in the horse, no descriptions of absent IPs in humans have been published. This may be explained by the very special contour of the equine teeth. Nevertheless, the assessment of the contour of the equine gingiva might serve as a meaningful criterion to diagnose early phases of PD, although the entire gingiva is not accessible during routine dental examination.

## Conclusions

The anatomical description of the healthy equine gingiva creates a basis for further research into the prevention and early detection of gingival and periodontal diseases. It provides detailed anatomical descriptions for the gingival sulcus, periodontal pockets, diastemata, mucogingival junction, interdental papillae, and the shape and contour of the gingival margin, which are necessary for the correct diagnoses of (early) pathological alterations.

## Data Availability Statement

The datasets generated for this study are available on request to the corresponding author.

## Ethics Statement

Ethical review and approval was not required for the animal study because 20 horses of different breeds, aged from <9 months to 26 years, were euthanized for reasons not related to this study. Written informed consent was obtained from the owners for the participation of their animals in this study.

## Author Contributions

CS and MR contributed conception and design of the study. KF performed the statistical analysis. JV organized the database. All authors contributed to manuscript revision, read, and approved the submitted version.

### Conflict of Interest

The authors declare that the research was conducted in the absence of any commercial or financial relationships that could be construed as a potential conflict of interest.
